# The Translation and Cross-Cultural Adaptation of an Arabic Version of the Reminiscence Functions Scale

**DOI:** 10.1093/geroni/igaa057.1102

**Published:** 2020-12-16

**Authors:** Abdallah Abu Khait, Juliette Shellman

**Affiliations:** 1 University of Connecticut, Storrs, Connecticut, United States; 2 University of Connecticut, Storrs Mansfield, Connecticut, United States

## Abstract

The Reminiscence Functions Scale (RFS), a 43 item reliable and valid scale, measures eight specific reasons as to why individuals reminisce: (a) identity (b) death preparation; (c) problem-solving; (d) bitterness revival; (e) boredom reduction; (f) intimacy maintenance; (g) conversation; and (h) teach/Inform others. Research indicates that certain reminiscence functions have a positive impact on the mental-health and well-being of older adults. However, no known studies have been conducted in Arab countries examining the relationship between reminiscence functions and mental health outcomes due to the lack of an Arabic version of the RFS. The purpose of this study was to translate the RFS from English to Arabic (Modern Standard Arabic), back-translate from Arabic to English, and compare the two English versions for equivalence and accuracy through a multi-step translation method. A team of bilingual, bicultural, Arabic speaking experts assembled to conduct the forward, back translation and harmonization process. In the next step, professionals with expertise in linguistics communication sciences and disorders, Arabic literature, geriatric nursing, and medicine reviewed the translated documents to assess the content (relevant to the target culture) and semantic equivalencies (similarity of meaning in the target culture). Challenges that occurred during the study included finding nuanced translation equivalences for Likert scale responses, translation of idioms such as “when time is heavy on my hands”, and logistical issues such as coordinating virtual meetings for the team of experts. Lessons learned during the translation process and implications for use of the RFS-Arabic version with Jordanian older adults will be presented.

